# Socioeconomic risk markers of congenital Zika syndrome: a nationwide, registry-based study in Brazil

**DOI:** 10.1136/bmjgh-2022-009600

**Published:** 2022-09-29

**Authors:** Enny S Paixão, Qeren Hapuk R Ferreira Fernandes, Luciana L Cardim, Julia M Pescarini, Maria Conceicao N Costa, Ila R Falcão, Elizabeth B Brickley, Andreia Costa Santos, André Portela Souza, Rita de Cassia Oliveira Carvalho-Sauer, Liam Smeeth, Laura C Rodrigues, Mauricio L Barreto, Maria Gloria Teixeira

**Affiliations:** 1 Department of Infectious Disease Epidemiology, London School of Hygiene & Tropical Medicine, London, UK; 2 Center of Data and Knowledge Integration for Health (CIDACS), Gonçalo Moniz Institute, Oswaldo Cruz Foundation, Salvador, Brazil; 3 Instituto de Saude Coletiva, Federal University of Bahia, Salvador, Brazil; 4 School of Economics and Center for Applied Microeconomic Studies, Getulio Vargas Foundation, São Paulo, Brazil; 5 East Regional Health Center of the State Health Secretariat of Bahia, Santo Antonio de Jesus, Brazil; 6 Faculty of Epidemiology and Population Health, London School of Hygiene and Tropical Medicine, London, UK

**Keywords:** public health, arboviruses, child health, epidemiology

## Abstract

While it is well known that socioeconomic markers are associated with a higher risk of arbovirus infections, research on the relationship between socioeconomic factors and congenital Zika syndrome (CZS) remains limited. This study investigates the relationship between socioeconomic risk markers and live births with CZS in Brazil. We conducted a population-based study using data from all registered live births in Brazil (Live Births Information System) linked with the Public Health Event Record from 1 January 2015 to 31 December 2018. We used logistic regression models to estimate the OR and 95% CIs of CZS based on a three-level framework. In an analysis of 11 366 686 live births, of which 3353 had CZS, we observed that live births of self-identified black or mixed race/brown mothers (1.72 (95% CI 1.47 to 2.01) and 1.37 (95% CI 1.24 to 1.51)) were associated with a higher odds of CZS. Live births from single women compared with married women and those from women with less than 12 years of education compared with those with more than 12 years of education also had higher odds of CZS. In addition, live births following fewer prenatal care appointments had increased odds of CZS in the nationwide data. However, in the analyses conducted in the Northeast region (where the microcephaly epidemic started before the link with Zika virus was established and before preventive measures were known or disseminated), no statistical association was found between the number of prenatal care appointments and the odds of CZS. This study shows that live births of the most socially vulnerable women in Brazil had the greatest odds of CZS. This disproportionate distribution of risk places an even greater burden on already socioeconomically disadvantaged groups, and the lifelong disabilities caused by this syndrome may reinforce existing social and health inequalities.

**WHAT IS ALREADY KNOWN ON THIS TOPIC**

- Socioeconomic markers are associated with a higher risk of arbovirus infections.
- Ecological studies suggest that the incidence of Zika virus (ZIKV) infections during pregnancy and congenital Zika syndrome (CZS) cases were more frequent in areas with poor living conditions at the neighbourhood level.

**WHAT THIS STUDY ADDS**

- Live births of self-identified black or mixed race/brown mothers, single women and those with less than 12 years of education had a higher odds of CZS.
- In the nationwide data, children with fewer prenatal care appointments were also associated with increased odds of CZS than those with six or more appointments. The highest odds were observed among those who did not attend prenatal visits. However, in the analyses conducted in the Northeast region (where the microcephaly epidemic started before the link with ZIKV was established and before preventive measures were known or disseminated), no statistical association was found between the number of prenatal care appointments and the odds of CZS.

**HOW THIS STUDY MIGHT AFFECT RESEARCH, PRACTICE OR POLICY**

- The disproportionate distribution of CZS in Brazil places an even more significant burden on already socioeconomically disadvantaged groups, and the lifelong disabilities caused by this syndrome may reinforce existing social and health inequalities.

## Introduction

Zika is a mosquitoborne viral disease that has attracted interest in recent years due to its expanding geographical range and harmful effects caused to developing fetuses when congenitally infected.^1–3^ Offspring with congenital Zika syndrome (CZS) can present with a broad spectrum of clinical manifestations that can be detected during pregnancy or after birth.^4 5^ Emerging evidence suggests that children with CZS have lifelong health sequelae that can increase mortality risk^6^ and require costly interventions with widespread social and economic impacts on the affected individual and their family.^7^ While it is well known that social risk markers at the community (eg, sanitation), household (eg, crowding, housing materials) and individual (eg, education) levels are associated with a higher risk of infectious diseases^4 8–13^ including arboviruses,^14^ research on the relationship between socioeconomic factors and CZS remains limited.

During the recent Zika virus (ZIKV) epidemic, Brazil experienced a record number of live births with CZS, with almost 90% of all confirmed cases worldwide.^15^ Of the CZS cases registered in Brazil between 2015 and 2018, more than 60% were born in the Northeast region, one of the country’s poorest areas—likely reflecting a combination of social and economic deprivation, inequalities in access to health services and environmental factors. Ecological studies suggest that the incidence of ZIKV infections during pregnancy^16^ and CZS cases were more frequent in areas with poor living conditions at the neighbourhood level.^17 18^

A better understanding of the social risk markers of CZS could inform targeted health strategies for preventive measures and promote early detection of the syndrome in high-risk populations. Using national population-based data collected from 2015 to 2018 in Brazil, this study investigates the relationship between socioeconomic risk markers and live births with CZS.

## Methods

### Study design and data source

We conducted a population-based study, including all registered live births in Brazil from 1 January 2015 to 31 December 2018, using data made available by the Brazilian Ministry of Health (MoH) and extracted in 2020.

The Live Births Information System (Sistema de Informação sobre Nascidos Vivos-SINASC)^19^ is an information system with 100% coverage of the country that records data from the Declaration of Live Births, a legal document completed by the health worker who assists the delivery.^20^ From this system, we obtained information about the mother (maternal age, schooling, marital status and race/ethnicity); the pregnancy (number of prenatal appointments, length of gestation, number of fetuses) and the newborn (birth weight and sex).

To identify those individuals with CZS, we obtained information from the Public Health Event Record (Registro de Eventos em em Saúde Pública, RESP),^21^ which registered information from all cases with suspected microcephaly and/or central nervous system (CNS) alterations possibly associated with congenital ZIKV infection since 2015. From RESP, we retained data on final CZS classification (ie, confirmed/probable, excluded or inconclusive).

### Linkage process

Live births records from SINASC were linked with RESP using the name, age and place of residence of the mother as matching variables. The linkage was performed with Centro de Integracao de Dados e Conhecimentos para a Saúde (CIDACS)-Record Linkage, a record-linkage tool developed to link large-scale administrative datasets applying the combination of indexing and searching algorithms approach.^22^ All linkage procedures were conducted in a strict data protection environment and according to ethical and legal rules from the Centre of Data and Knowledge Integration for Health (CIDACS).^23^ After linkage, the data were deidentified and analysed in a virtual machine accessed via a virtual private network, without external access to internet.

### Procedures

According to the Brazilian MoH, live births should be reported and investigated as suspected cases of CZS if they present with at least one of the following criteria:

1. Microcephaly, defined as head circumference (HC) of 33 cm or less for term (ie, ≥37 weeks gestation) boys and girls, which was reduced to ≤32 cm on 12 December 2015, and reduced again in March 2016, following the WHO recommendation, to ≤31.9 cm for term boys and ≤31.5 cm for term girls or more than 2 SDs below the mean for age and sex, according to INTERGROWTH 21st standards for preterm births, or WHO standards for term births;
2. Craniofacial disproportion; CNS changes suggestive of congenital infection detected from neuroimaging tests; two or more neurological, visual or auditory manifestations, such as ventriculomegaly, hydrocephalus, dysgenesis of the corpus callosum, microphthalmia, partial or complete atrophy of the optic nerve, injuries in the retina, hearing loss, among others and
3. Newborns or fetuses from mothers who had fever and skin rash during pregnancy, likely or confirmed for ZIKV infection, regardless of the identification of congenital malformations at birth.^24 25^

After notification, all suspected cases were investigated by local epidemiological surveillance teams and classified as^25^:

1. Confirmed/probable, if they had signs and symptoms consistent with CZS regardless of laboratory confirmation or maternal symptoms;
2. Excluded, if they had compatible clinical symptoms that, after clinical and epidemiological investigation, were attributed to having another cause; for example, microcephaly related to restricted intrauterine growth or genetic diseases or
3. Inconclusive, if there was insufficient information for proper classification.

This study included all live births during the study period, defined as the primary study outcome cases of confirmed or probable CZS linked with live births. We excluded (1) suspected CZS cases still under investigation or found to be inconclusive and^26^ live births with congenital anomalies registered in SINASC but without CZS classification.

### Statistical analyses

**Figure 1:**
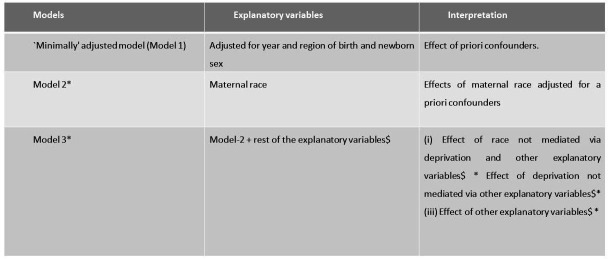
Concptual framework for the association of sociodemograpic factors with conginital Zika syngrome. *All variables added in the model adjusted for each other and priori confounders: year and region of birth and sex. $Maternal age, maternal education, maternal marital status, number of prenatal care appointments.

We estimated the crude prevalence of CZS in all individuals with available data for each potential socioeconomic risk markers as well as described patterns of missing data. Based on the literature,^4 8–13^ we developed a framework to investigate the social determinants of CZS in Brazil (figure 1). The framework defined three levels, which included model 1: a minimally adjusted model for the effect of year of birth, Brazilian region of birth and newborn sex on CZS; model 2: a model estimating the effect of maternal race/ethnicity (ie, as a proxy for systemic and structural/institutional racism^27^ adjusted for a priori confounders (year, region and sex) and model 3: a model estimating (1) the effect of race/ethnicity not mediated via other explanatory variables (maternal age, maternal education, maternal marital status, number of prenatal care appointments) and^26^ the effect of other explanatory variables. We used logistic regression models to estimate the adjusted OR and 95% CIs of CZS. All variables from previous models were included as explanatory variables in the next level model. We repeated the analyses for the Northeast region, since it concentrated over 60% of the CZS cases. Data analyses were performed in Stata V.17.0.

### Patient and public involvement

The patients and the public were not involved in the design, conduct or reporting of our research.

## Results

**Figure 2:**
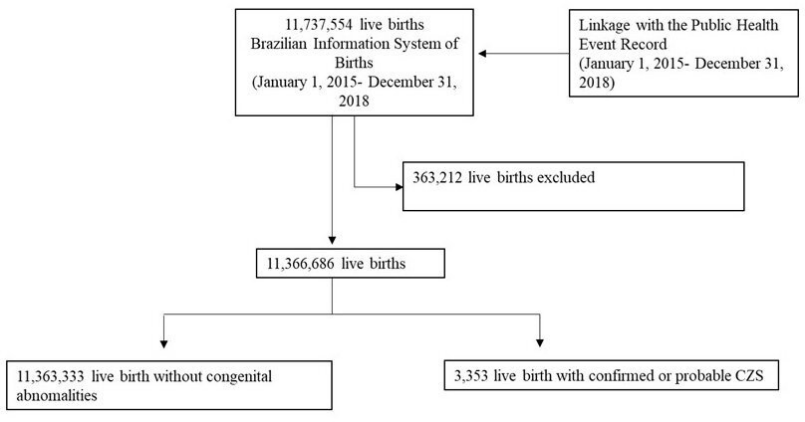
Flow chart stusy population Brazil 2015–2018. CZS, congenital Zika syndrome.

The study population included 11 366 686 live births (figure 2), of which 3353 had CZS. The highest prevalence of CZS per 100 000 live births was observed in 2016 (56.3; 95% CI 53.5 to 59.2), and the lowest prevalence in 2018 (6.9; 95% CI 6.0 to 8.0). The Northeast region experienced the highest burden of cases, with a prevalence of 63.4/100 000 live births (95% CI 60.6 to 66.1), followed by the Central-West, with a prevalence of 30.3 (95% CI 26.8 to 34.1). The South region had the lowest prevalence, with 4.7/100 000 live births. The prevalence of CZS registration was almost 20% higher among female vs male newborns. Based on crude frequencies, children born with CZS were more likely to have mothers who were Black or mixed race/Brown, less educated, single, younger and/or who received fewer prenatal appointments (table 1).

**Table 1:** Baseline characteristics of the study population

Demographic variables	No of individuals with missing data	No of individuals	No of congenital Zika syndrome cases	Prevalence × 100 000 (95% CI)
Year of birth	0	–	–	–
2015	–	2 913 812 (25.6%)	1226	42.1 (39.8 to 44.5)
2016	–	2 769 476 (24.4%)	1560	56.3 (53.6 to 59.2)
2017	–	2 829 285 (24.9%)	369	13.0 (11.8 to 14.4)
2018	–	2 854 113 (25.1%)	198	6.9 (6.0 to 8.0)
Sex of the newborn	11 (<0.1%)	–	–	–
Male	–	5 818 371 (51.2%)	1566	26.9 (25.6 to 28.3)
Female	–	5 548 304 (48.8%)	1779	32.1 (30.6 to 33.6)
Birth region	3511 (<0.1%)	–	–	–
South	–	1 567 773 (13.8%)	74	4.7 (3.7 to 5.9)
Southeast	–	4 496 224 (39.6%)	813	18.1 (16.9 to 19.4)
North	–	1 230 977 (10.8%)	185	15.0 (12.9 to 17.4)
Northeast	–	3 172 975 (27.9%)	2010	63.4 (60.6 to 66.2)
Midwest	–	895 226 (7.9%)	271	30.3 (26.8 to 34.1)
Maternal ethnicity	461 094 (4.1%)	–	–	–
White	–	4 004 683 (35.2%)	593	14.8 (13.6 to 16.1)
Black	–	606 749 (5.3%)	227	37.4 (32.7 to 42.6)
Mixed race/brown	–	6 157 534 (54.2%)	2293	37.2 (35.7 to 38.8)
Asian	–	43 912 (0.4%)	12	27.3 (14.1 to 47.7)
Indigenous	–	92 714 (0.8%)	20	21.6 (13.2 to 33.3)
Maternal education (Years)	149 407 (1.3%)	–	–	–
1–3	–	314 986 (2.8%)	132	41.9 (35.1 to 49.7)
4–7	–	1 904 078 (16.8%)	762	40.0 (37.2 to 43.0)
12–8	–	6 768 809 (59.5%)	2034	30.1 (28.8 to 31.4)
>12	–	2 229 406 (19.6%)	372	16.7 (15.0 to 18.5)
Maternal marital status	111 412 (1.0%)	–	–	–
Married/union	–	6 268 759 (55.2%)	1555	24.8 (23.6 to 26.1)
Single		4 833 219 (42.5%)	1724	35.7 (34.0 to 37.4)
Widow		19 506 (0.2%)	6	30.8 (11.3 to 66.9)
Divorced	–	133 790 (1.2%)	28	20.9 (13.9 to 30.3)
No of prenatal appointments	283 429 (2.5%)	–	–	–
None	–	63 847 (0.6%)	52	81.4 (60.8 to 106.8)
1–3	–	673 788 (6.1%)	307	45.6 (40.6 to 51.0)
4–5	–	1 370 981 (12.4%)	534	38.9 (35.7 to 42.4)
6+	–	8 974 641 (80.9%)	2328	25.9 (24.9 to 27.0)
Maternal age (years)	139 (<0.1%)	–	–	–
<20	–	1 920 351 (16.9%)	776	40.4 (37.6 to 43.4)
20–34	–	7 850 607 (69.1%)	2205	28.1 (26.9 to 29.3)
35+	–	1 595 589 (14.0%)	372	23.3 (21.0 to 25.8)

In the minimally adjusted model 1, live births from mothers living in the Northeast region had approximately 13.5 times higher odds of being a CZS case than mothers living in the South region (table 2). After adjusting for year and region of birth and newborn sex in model 2, the odds of CZS were 1.72 (95% CI 1.47 to 2.01) and 1.37 (95% CI 1.24 to 1.51) higher among live births of mothers who self-identified as black or mixed race/brown than those identifying as white. In the fully adjusted model 3, other explanatory variables attenuated the association between race/ethnicity and CZS; however, live births from black (1.46; 95% CI 1.25 to 1.73) or mixed race/brown (1.21; 95% CI 1.09 to 1.34) mothers still had increased odds of CZS. The odds of CZS were up to 30% higher comparing live births of women with less than 12 years of education to those with more than 12 years of education. Single women had 1.38 times (95% CI 1.28 to 1.49) higher odds of having a live birth with CZS than married women. Live births following fewer prenatal care appointments were also associated with increased odds of CZS compared with those with six or more appointments: the odds were 1.14 times (95% CI 1.04 to 1.26) higher for those with 4–5 visits, 1.34 times (95% CI 1.18 to 1.51) higher for those with 1–3 visits and 2.82 times (95% CI 2.10 to 3.77) higher for those who did not attend prenatal visits.

**Table 2:** Multivariate association of socioeconomic factors with CZS, Brazil 2015–2018

Demographic variables	Model 1*		Model 2†		Model 3‡	
	OR (95% CI)	P value	OR (95% CI)	P value	OR (95% CI)	P value
Year of birth						
2015	1		1		1	
2016	1.34 (1.25 to 1.45)	<0.001	1.31 (1.21 to 1.42)	<0.001	1.29 (1.20 to 1.40)	<0.001
2017	0.31 (0.28 to 0.35)	<0.001	0.30 (0.27 to 0.34)	<0.001	0.29 (0.26 to 0.33)	<0.001
2018	0.16 (0.14 to 0.19)	<0.001	0.16 (0.14 to 0.19)	<0.001	0.15 (0.13 to 0.18)	<0.001
Sex of the newborn						
Male	1		1		1	
Female	1.19 (1.11 to 1.28)	<0.001	1.19 (1.11 to 1.28)	<0.001	1.20 (1.11 to 1.28)	<0.001
Birth region						
South	1		1		1	
Southeast	3.83 (3.02 to 4.86)	<0.001	3.40 (2.66 to 4.33)	<0.001	3.41 (2.67 to 4.37)	<0.001
North	3.19 (2.44 to 4.18)	<0.001	2.66 (2.01 to 3.53)	<0.001	2.71 (2.03 to 3.61)	<0.001
Northeast	13.51 (10.72 to 17.04)	<0.001	11.07 (8.66 to 14.13)	<0.001	11.36 (8.88 to 14.54)	<0.001
Central-west	6.47 (5.00 to 8.37)	<0.001	5.43 (4.15 to 7.10)	<0.001	5.68 (4.32 to 7.45)	<0.001
Maternal race/ethnicity						
White			1		1	
Black			1.72 (1.47 to 2.01)	<0.001	1.46 (1.25 to 1.73)	<0.001
Mixed brown			1.37 (1.24 to 1.51)	<0.001	1.21 (1.09 to 1.34)	<0.001
Asian			1.33 (0.75 to 2.36)	0.329	1.33 (0.75 to 2.36)	0.327
Indigenous			1.19 (0.76 to 1.87)	0.442	0.98 (0.61 to 1.57)	0.929
Maternal education (years)						
0–3					1.20 (0.97 to 1.50)	0.094
4–7					1.30 (1.13 to 1.50)	<0.001
12–8					1.28 (1.13 to 1.44)	<0.001
> 12					1	
Maternal marital status						
Married/union					1	
Single					1.38 (1.28 to 1.49)	<0.001
Widow					0.88 (0.33 to 2.34)	0.794
Divorced					1.13 (0.75 to 1.69)	0.557
No of prenatal appointments						
None					2.82 (2.10 to 3.77)	<0.001
1–3					1.34 (1.18 to 1.51)	<0.001
4–5					1.14 (1.04 to 1.26)	0.006
6+					1	
Maternal age (years)						
<20					1.12 (0.97 to 1.28)	0.116
20–34					1.02 (0.90 to 1.15)	0.773
35+					1	

*Model 1 is a minimally adjusted model—adjusted for year of birth, region and sex of the newborn.

†Model 2—year of birth, region and sex and race.

‡Model 3—year of birth, region and sex, race, maternal education, marital status, maternal age, number of prenatal appointments.

CZS, congenital Zika syndrome.

In the analyses restricted to the Northeast region and adjusted for year and newborn sex, similar to the previous nationwide model, live births of mothers who self-declared to be black had 1.72 times (95% CI 1.39 to 2.12) higher odds of CZS, compared with live births of white women (table 3). In the fully adjusted model, live births of women with 8–12 years of education had 27% higher odds of having a live birth with CZS than of women with more than 12 years of education, although no significant differences were observed in the other categories of education. Single women had 1.30 times (95% CI 1.18 to 1.43) higher odds of having a live birth with CZS than married women. However, there was no significant association between the number of prenatal care appointments and the chance of CZS in this region.

**Table 3:** Multivariate association of socioeconomic factors with CZS in Northeast Brazil 2015–2018

Demographic variables	Model 1*		Model 2†		Model 3‡	
	OR (95% CI)	P value	OR (95% CI)	P value	OR (95% CI)	P value
Year of birth						
2015	1		1		1	
2016	0.71 (0.65 to 0.79)	<0.001	0.70 (0.63 to 0.78)	<0.001	0.68 (0.62 to 0.76)	<0.001
2017	0.14 (0.12 to 0.17)	<0.001	0.14 (0.11 to 0.16)	<0.001	0.13 (0.11 to 0.16)	<0.001
2018	0.09 (0.07 to 0.11)	<0.001	0.09 (0.07 to 0.11)	<0.001	0.08 (0.06 to 0.10)	<0.001
Sex of the newborn						
Male	1		1		1	
Female	1.22 (1.11 to 1.33)	<0.001	1.23 (1.12 to 1.34)	<0.001	1.20 (1.10 to 1.32)	<0.001
Maternal ethnicity						
White			1		1	
Black			1.72 (1.39 to 2.12)	<0.001	1.56 (1.25 to 1.94)	<0.001
Mixed Brown			1.12 (0.97 to 1.30)	0.130	1.04 (0.89 to 1.21)	0.639
Asian			0.39 (0.10 to 1.59)	0.190	0.39 (0.10 to 1.59)	0.190
Indigenous			1.01 (0.50 to 2.05)	0.979	0.89 (0.42 to 1.90)	0.749
Maternal education (years)						
0–3					1.19 (0.92 to 1.54)	0.198
4–7					1.16 (0.97 to 1.40)	0.130
12–8					1.27 (1.08 to 1.51)	0.005
>12					1	
Maternal marital status						
Married/union					1	
Single					1.30 (1.18 to 1.43)	<0.001
Widow					0.70 (0.17 to 2.79)	0.609
Divorced					1.10 (0.57 to 2.12)	0.782
No of prenatal appointments						
None					1.24 (0.68 to 2.25)	0.476
1–3					1.08 (0.90 to 1.27)	0.350
4–6					1.00 (0.88 to 1.14)	0.894
7+					1	
Maternal age (years)						
<20					1.05 (0.88 to 1.25)	0.621
20–34					0.91 (0.78 to 1.06)	0.218
35+					1	

*Model 1 is a minimally adjusted model—adjusted for year of birth, region and sex of the newborn.

†Model 2 year of birth, region and sex and race.

‡Model 3—year of birth, region and sex, race, maternal education, marital status, maternal age, number of prenatal appointments.

CZS, congenital Zika syndrome.

## Discussion

In this nationwide study of more than 11 million births and 3353 cases of CZS, we found evidence that the most socially vulnerable group of live births in Brazil had the greatest risks of having a newborn with CZS. Live births of mothers in the Northeast region, one of the regions with the most widespread poverty in the country (Northeast),^28^ had greater CZS odds than mothers from the South region. Similarly, live births of self-identified black or mixed race/brown mothers were associated with a higher odds of CZS, even after adjusting for several socioeconomic and demographic characteristics. In addition, live births from single women showed 37% higher odds of CZS. Notably, live births following fewer prenatal care appointments had increased odds of CZS in the nationwide data. The findings in the analysis restricted to the Northeast region showed similar results, except that no evidence of a statistical association was found between the number of prenatal care appointments and the odds of CZS.

Our study found markers of social vulnerability, such as living in the Northeast, the mother’s race/ethnicity, years of education and marital status, to be associated with a higher risk of CZS. The findings are broadly consistent with a 2022 systematic review of socioeconomic risk factors for arbovirus infections that found an association between lower education, income poverty, low healthcare coverage, poor housing materials, interrupted water supply, non-white ethnicities and migration status and arbovirus infection.^14^ Indicators of low socioeconomic position associated with increased CZS risks may be explained, in part, by increased exposure to infected mosquitoes during pregnancy due to community-level environmental factors such as interrupted water supplies/sanitation and inadequate housing conditions. A literature review on infectious diseases in Brazil published in 2011,^29^ had already stressed the failure of the control of *Aedes aegypti* in the country, which at that time was responsible for major dengue epidemics in the country and is the main vector of ZIKV. The lack of affordable mosquito repellent and awareness of how to avoid mosquito bites can also explain this association. Another pathway that could explain this association could be disparities in access to care, including diagnosis before birth and options for termination of pregnancy once the fetus is diagnosed during pregnancy.

This study provides important evidence of racial disparities in CZS risks, such that black and mixed-race/brown women had increased odds of having a live birth with CZS than their white counterparts after adjustment for year, newborn sex, region, maternal education, maternal age, maternal marital status and the number of prenatal care visits. For this analysis, we conceptualised maternal race/ethnicity as a proxy for systemic and structural racism. Systemic and structural racism can drive racial inequities in health outcomes through various causal pathways.^27^ Embedded discrimination can lead to systemic disadvantages; for example, within the Brazilian economic system, individuals who are black are three times more likely to live in extreme poverty.^30^ In addition, structural racism (eg, as manifested in policies) may impose institutional barriers for non-white population groups to access resources and opportunities, resulting in lower levels of education, poorer living conditions and inadequate health care.^31^ Within the maternal–child health context, institutional racism can result in racial inequities in access and quality of prenatal and perinatal care, including access to maternal interventions including pregnancy terminations.^32^

Previous studies have demonstrated that single women are more likely to give birth to newborns with adverse pregnancy outcomes, such as small for gestational age, when compared with married mothers.^33^ Being single is increasingly recognised as a risk factor for adverse pregnancy outcomes, potentially due to a lack of social support or increased stress.^34^ In addition, the prevalence of CZS was higher among younger women, and they are more likely to be single. Reverse causality may also be an important explanation for the association between single parents and CZS; for example, there is some evidence suggesting that ending of a civil relationship or divorce may be more likely following a fetal CZS diagnosis during pregnancy.^35^

A dose–response association was observed between the number of prenatal visits and CZS; the fewer prenatal care visits, the higher the odds of CZS. The lack of prenatal care or insufficient appointments has been associated with adverse pregnancy outcomes,^36–39^ such as small for gestational age. The primary prevention of ZIKV infections during pregnancy is conditional on the use of insect repellent to prevent mosquito-borne transmission and condoms to prevent sexual transmission.^40^ The effectiveness of these protective measures has been associated with receiving direct guidance from health professionals during prenatal care visits. Studies have shown that the cost of insect repellent is another obstacle to its use.^41^ Therefore, prenatal care, including education and free distribution of preventive items (eg, insect repellent), are likely to lead to more effective prevention of ZIKV infections during pregnancy, especially for populations with low financial resources.^40^ Of note, the lack of association between the number of prenatal care appointments and CZS in the Northeast region is likely due to the fact that the microcephaly epidemic started in this region prior to the causal association being established with ZIKV. Due to the earlier peak of the ZIKV epidemic in this region, we hypothesise that pregnant populations in the Northeast would therefore have been less likely to be informed about preventive measures during prenatal visits.

The Northeast region of Brazil concentrates almost half of the Brazilian population living in poverty,^42^ and it was where the majority of CZS cases have occurred since 2015. An ecological study in Recife, the capital of the State of Pernambuco, in North-eastern Brazil, suggested an association between neighbourhood-level income and ZIKV infection risks in pregnant people.^16^ Another ecological study also conducted in Recife found microcephaly cases were up to 5.6 times more prevalent in districts of low socioeconomic status than those of high socioeconomic status.^17^ Similarly, a cross-sectional hospital-based study in Salvador, Bahia state, found that lower education and food insecurity were associated with ZIKV exposure among pregnant persons.^11^ In the Southeast region of Brazil, a prospective cohort study of ZIKV during pregnancy in Rio de Janeiro also found higher risks of microcephaly among children born to families with lower household income, lower maternal education, and greater household crowding.^43^ Conversely, a small case-control study of mothers exposed to ZIKV during pregnancy in Rio de Janeiro found no differences in CZS risk according to the income of the mothers.^44^ A study carried out in Colombia found a negative association between ZIKV reporting and unsatisfied basic needs in the population level.^13^

A strength of our study was the large nationwide sample size, including all confirmed and probable CZS cases notified in the country of Brazil. We also included a population-representative comparison group. There are, however, limitations. First, this study was based on registry data, and relevant, more detailed information such as family income and household crowding were not available. Second, there may have been underreporting in the RESP, mainly among those fetuses prenatally exposed to ZIKV, but without detectable malformations at birth. Third, the changing case definition of microcephaly can generate biased estimation. For example, the first case definition of microcephaly did not distinguish between sex and may lead to overestimating the frequency of microcephaly among live female births. Finally, the linkage process could have introduced classification bias due to a linkage error.

## Conclusion

This study shows that live births of the most socially vulnerable women in Brazil are at the greatest chance of CZS, particularly those living in the Northeast region, who identify as black or mixed race/brown, single, with lower number of years of education and/or lower prenatal healthcare access. This disproportionate distribution of risk places an even greater burden on already socioeconomically disadvantaged groups and the lifelong disabilities caused by this syndrome may reinforce existing social and health inequalities. An intersectional analysis is warranted to see the effects of multiple social identities. It is also important to evaluate the importance of social protection policies as potential mitigators of the deleterious impacts of socioeconomic risk markers of CZS. Our results have important implications for facilitating the detection of high-risk women and reinforcing the importance of prenatal care and preventive measures such as the use of repellents to avoid mosquito biting and condoms to prevent sexually transmitted Zika during pregnancy. Community measures such as improved sanitation can also effectively reduce the mosquito population. Therefore, strategies aiming to increase access to healthcare and sanitation in the poorest populations might benefit from improving primary prevention against *Aedes mosquito* bites and ZIKV infections during pregnancy.

10.1136/bmjgh-2022-009600.supp1 Supplementary data

## Data Availability

Data are available on reasonable request. The data that support the findings of this study are available from Brazilian Ministry of Health, but restrictions apply to the availability of these data, which were used under licence for the current study, and so are not publicly available. Data are, however available from the authors upon reasonable request and with permission of the Brazilian Ministry of Health.

## References

[1] Lessler J, Chaisson LH, Kucirka LM, et al. Assessing the global threat from Zika virus. Science 2016;353:aaf8160. 10.1126/science.aaf8160 27417495 PMC5467639

[2] Musso D, Gubler DJ. Zika virus. Clin Microbiol Rev 2016;29:487–524. 10.1128/CMR.00072-15 27029595 PMC4861986

[3] PAHO/WHO. Regional Zika epidemiological update (Americas) August 25, 2017, 2017. Available: https://www.paho.org/hq/dmdocuments/2017/2017-aug-25-phe-epi-update-zika-virus.pdf

[4] Faria NR, Azevedo RdoSdaS, Kraemer MUG, et al. Zika virus in the Americas: early epidemiological and genetic findings. Science 2016;352:345–9. 10.1126/science.aaf5036 27013429 PMC4918795

[5] Honein MA, Dawson AL, Petersen EE, et al. Birth defects among fetuses and infants of US women with evidence of possible Zika virus infection during pregnancy. JAMA 2017;317:59–68. 10.1001/jama.2016.19006 27960197

[6] Paixao ES, Cardim LL, Costa MCN, et al. Mortality from congenital zika syndrome - Nationwide cohort study in Brazil. N Engl J Med 2022;386:757–67. 10.1056/NEJMoa2101195 35196428 PMC7612437

[7] Duttine A, Smythe T, Ribiero Calheiro de Sá M, et al. Congenital Zika Syndrome-Assessing the need for a family support programme in Brazil. Int J Environ Res Public Health 2020;17:17103559. 10.3390/ijerph17103559 PMC7277658 32438700

[8] Morano JP, Holt DA. The social determinants of health contextualized for the Zika virus. Int J Infect Dis 2017;65:142–3. 10.1016/j.ijid.2017.10.006 29081365

[9] Ferguson NM, Cucunubá ZM, Dorigatti I, et al. Epidemiology. Countering the Zika epidemic in Latin America. Science 2016;353:353–4. 10.1126/science.aag0219 27417493 PMC5475255

[10] Ali S, Gugliemini O, Harber S, et al. Environmental and social change drive the explosive emergence of Zika virus in the Americas. PLoS Negl Trop Dis 2017;11:e0005135. 10.1371/journal.pntd.0005135 28182667 PMC5300271

[11] Nery N, Aguilar Ticona JP, Gambrah C, et al. Social determinants associated with Zika virus infection in pregnant women. PLoS Negl Trop Dis 2021;15:e0009612. 10.1371/journal.pntd.0009612 34329305 PMC8323902

[12] Raymundo CE, de Andrade Medronho R. Association between socio-environmental factors, coverage by family health teams, and rainfall in the spatial distribution of Zika virus infection in the city of Rio de Janeiro, Brazil, in 2015 and 2016. BMC Public Health 2021;21:1199. 10.1186/s12889-021-11249-y 34162338 PMC8220830

[13] Rees EE, Petukhova T, Mascarenhas M, et al. Environmental and social determinants of population vulnerability to Zika virus emergence at the local scale. Parasit Vectors 2018;11:290. 10.1186/s13071-018-2867-8 29739467 PMC5941591

[14] Power GM, Vaughan AM, Qiao L, et al. Socioeconomic risk markers of arthropod-borne virus (arbovirus) infections: a systematic literature review and meta-analysis. BMJ Glob Health 2022;7:e007735. 10.1136/bmjgh-2021-007735 PMC9014035 35428678

[15] MdFPMd A, WVd S, Araújo TVB. Epidemia de microcefalia E vírus Zika: a construção do conhecimento em epidemiologia. Cad Saúde Pública 2018;34. 10.1590/0102-311X00069018 30328996

[16] Lobkowicz L, Power GM, De Souza WV, et al. Neighbourhood-level income and Zika virus infection during pregnancy in Recife, Pernambuco, Brazil: an ecological perspective, 2015-2017. BMJ Glob Health 2021;6:e006811. 10.1136/bmjgh-2021-006811 PMC8640636 34857522

[17] Souza WVde, Albuquerque MdeFPMde, Vazquez E, et al. Microcephaly epidemic related to the Zika virus and living conditions in Recife, northeast Brazil. BMC Public Health 2018;18:130. 10.1186/s12889-018-5039-z 29329574 PMC5767029

[18] Netto EM, Moreira-Soto A, Pedroso C, et al. High Zika virus seroprevalence in Salvador, northeastern Brazil limits the potential for further outbreaks. mBio 2017;8:17. 10.1128/mBio.01390-17 PMC5686533 29138300

[19] (cidade) SP. Declaração de Nascido Vivo. Manual de preenchimento da Declaração de Nascido Vivo In: CEInfo SMdSCdEeI, ed. São Paulo: Secretaria Municipal da Saúde, 2011.

[20] EdA B, Vico ESR, Md F. Cobertura, completude E confiabilidade das informações do Sistema de Informações sobre Nascidos Vivos de maternidades dA rede pública no município de São Paulo, 2011. Epidemiologia e Serviços de Saúde 2018;27. 10.5123/s1679-49742018000100011 29451610

[21] (RESP) MdSdBRdEeSP. Monitoramento integrado de vigilância E atenção saúde de condições relacionadas S infecções durante a gestação, identificadas no pré-natal, parto E puericultura. 2017, 2022. Available: http://www.resp.saude.gov.br/microcefalia#/painel [Accessed 2 Mar 2022].

[22] Barbosa GCG, Ali MS, Araujo B, et al. CIDACS-RL: a novel indexing search and scoring-based record linkage system for huge datasets with high accuracy and scalability. BMC Med Inform Decis Mak 2020;20:289. 10.1186/s12911-020-01285-w 33167998 PMC7654019

[23] Barreto ML, Ichihara MY, Almeida BA, et al. The centre for data and knowledge integration for health (CIDACS): linking health and social data in Brazil. Int J Popul Data Sci 2019;4:1140. 10.23889/ijpds.v4i2.1140 34095542 PMC8142622

[24] MdSd B. Orientações integradas de vigilância e atenção saúde no âmbito da Emergência de Saúde Pública de Importância Nacional: procedimentos para o monitoramento das alterações no crescimento e desenvolvimento a partir da gestação até a primeira infância, relacionadas infecção pelo vírus Zika e outras etiologias infeciosas dentro da capacidade operacional do SUS [recurso eletrônico]. In: Saúde SdVeSSdA, ed. Brasília Ministério da Saúde, 2017.

[25] MdSd B. Protocolo de atenção saúde e resposta ocorrência de microcefalia relacionada infecção pelo vírus zika [recurso eletrônico]. In: Saúde SdA, ed. Brasília: Ministério da Saúde, 2016.

[26] Stringhini S, Carmeli C, Jokela M, et al. Socioeconomic status and the 25 × 25 risk factors as determinants of premature mortality: a multicohort study and meta-analysis of 1·7 million men and women. Lancet 2017;389:1229–37. 10.1016/S0140-6736(16)32380-7 28159391 PMC5368415

[27] Braveman PA, Arkin E, Proctor D, et al. Systemic and structural racism: definitions, examples, health damages, and approaches to dismantling. Health Aff 2022;41:171–8. 10.1377/hlthaff.2021.01394 35130057

[28] LEdS S. A pobreza entre regiões no Brasil: uma análise através da abordagem multidimensional. [Monografia (Trabalho de Conclusão de Curso)]. Universidade Federal de Pernambuco, 2018.

[29] Barreto ML, Teixeira MG, Bastos FI, et al. Successes and failures in the control of infectious diseases in Brazil: social and environmental context, policies, interventions, and research needs. Lancet 2011;377:1877–89. 10.1016/S0140-6736(11)60202-X 21561657

[30] Osorio RG. A desigualdade racial da pobreza no Brasil. In: Ipea IdPEA, ed. Texto para discussão Brasília, Rio de Janeiro: Ipea, 2019.

[31] Werneck J. Institutional racism and black population health. Saúde e Sociedade 2016;25:535–49.

[32] Freitas Goes E, de Souza Menezes GM, Chagas de Almeida MdaC, et al. Barriers in accessing care for consequence of unsafe abortion by black women: evidence of institutional racism in Brazil. J Racial Ethn Health Disparities 2021;8:1385–94. 10.1007/s40615-020-00900-w 33439462

[33] Falcão IR, Ribeiro-Silva RdeC, de Almeida MF, et al. Factors associated with small- and large-for-gestational-age in socioeconomically vulnerable individuals in the 100 million Brazilian cohort. Am J Clin Nutr 2021;114:109–16. 10.1093/ajcn/nqab033 33826704 PMC8246620

[34] Lurie S, Zalmanovitch A, Golan A, et al. The effect of marital status on pregnancy outcome in Israel: a retrospective case-control study. J Obstet Gynaecol Res 2010;36:1161–4. 10.1111/j.1447-0756.2010.01312.x 21083838

[35] Smythe T, Duttine A, Vieira ACD, et al. Engagement of fathers in parent group interventions for children with congenital Zika syndrome: a qualitative study. Int J Environ Res Public Health 2019;16:3862. 10.3390/ijerph16203862 PMC6843372 31614765

[36] Dubois L, Girard M. Determinants of birthweight inequalities: population-based study. Pediatr Int 2006;48:470–8. 10.1111/j.1442-200X.2006.02256.x 16970785

[37] Minuci EG, Almeida MFde. [Birth weight intra-urban differentials in the city of São Paulo]. Rev Saude Publica 2009;43:256–66. 10.1590/s0034-89102009005000011 19225689

[38] Mumbare SS, Maindarkar G, Darade R, et al. Maternal risk factors associated with term low birth weight neonates: a matched-pair case control study. Indian Pediatr 2012;49:25–8. 10.1007/s13312-012-0010-z 21719926

[39] Falcão IR, Ribeiro-Silva RdeC, de Almeida MF, et al. Factors associated with low birth weight at term: a population-based linkage study of the 100 million Brazilian cohort. BMC Pregnancy Childbirth 2020;20:536. 10.1186/s12884-020-03226-x 32928144 PMC7491100

[40] Ramisetty-Mikler S, Boyce L. Communicating the risk of contracting Zika virus to low income underserved pregnant Latinas: a clinic-based study. PLoS One 2020;15:e0241675. 10.1371/journal.pone.0241675 33216763 PMC7679023

[41] Mendoza C, Jaramillo G-I, Ant TH, et al. An investigation into the knowledge, perceptions and role of personal protective technologies in Zika prevention in Colombia. PLoS Negl Trop Dis 2020;14:e0007970. 10.1371/journal.pntd.0007970 31961867 PMC7010294

[42] Governo do Estado do Ceará B. Região Nordeste possui quase metade de Toda a pobreza no Brasil, segundo IBGE: Secretaria do Planejamento E Gestão, 2020. Available: https://www.fecop.seplag.ce.gov.br/2020/11/20/regiao-nordeste-possui-quase-metade-de-toda-a-pobreza-no-brasil-segundo-ibge/

[43] Power GM, Francis SC, Sanchez Clemente N, et al. Examining the association of socioeconomic position with microcephaly and delayed childhood neurodevelopment among children with prenatal Zika virus exposure. Viruses 2020;12:v12111342. 10.3390/v12111342 PMC7700457 33238584

[44] Lima GP, Rozenbaum D, Pimentel C, et al. Factors associated with the development of congenital Zika syndrome: a case-control study. BMC Infect Dis 2019;19:277. 10.1186/s12879-019-3908-4 30902046 PMC6431070

