# Supplementary material for: Socioeconomic risk markers of congenital Zika syndrome: a nationwide, registry-based study in Brazil
Source: BMJ Glob Health. 2022 Sep 29;7(9):e009600. doi: 10.1136/bmjgh-2022-009600 (PMC9528618; doi:10.1136/bmjgh-2022-009600)
Supplement: Supplementary data [file bmjgh-2022-009600supp001.pdf]

Domain	Guiding questions	Author's response
Study conceptualisation	1. How does this study address local research and policy priorities?<br>2. How were local researchers involved in study design?	Brazil is the country that concentrated almost 90% of all confirmed cases of congenital zika syndrome worldwide. The emergence of such cases mobilized several actors in Brazil and worldwide to understand their cause, as well as their impact on children and families. Therefore, understanding social risk markers related to this syndrome is not only a local policy priority but can also be used by other affected countries.<br>This study is part of collaborative work between the CIDACS and the Brazilian Ministry of Health. Most of the authors are Brazilian, including the first and last author. The international collaborators invited to be co-authors are either experts in using electronic health records (LS) or have experience with Congenital Zika Syndrome (EBB). Both LS and EBB have had sustained partnerships with Brazilian researchers.
Research management	1. How has funding been used to support the local research team(s)?	The Brazilian Ministry of health locally provided the primary funding for this project. ESP, the first author is a Brazilian researcher who holds a Wellcome Trust award.
Data acquisition and analysis	1. How are research staff who conducted data collection acknowledged?<br>2. How have members of the research partnership been provided with access to study data?<br>3.How were data used to develop analytical skills within the partnership?	This study used secondary data provided by the Brazilian Ministry of Health and linked by the CIDACS team. Both teams acknowledged this in the manuscript.<br>These data have been used for several studies conducted in Brazil to develop the analytical skills of PhD students and early career researchers, some of them included in this paper.
Data interpretation	1. How have research partners collaborated in interpreting study data?	The researchers from LSHTM and the CIDACS Zika Platform meet every other week to discuss the findings of this and additional works conducted on the Zika Platform.
Drafting and revising for intellectual content	1. How were research partners supported to develop writing skills?<br>2. How will research products be shared to address local needs?	ESP, the first author of this study, wrote the first draft, and all co-authors provided critical review. These drafts circulated among all the authors several times, so early career researchers involved in the manuscript had the opportunity to learn writing skills during the

		process.<br>Because this study was developed as part of a bigger project, the researchers included in this project have scheduled several meetings to disseminate the findings. Studies developed by researchers on this platform have been used in guidelines produced by the Brazilian Ministry of Health.
Authorship	1. How is the leadership, contribution and ownership of this work by LMIC researchers recognised within the authorship?<br>2. How have early career researchers across the partnership been included within the authorship team?<br>3. How has gender balance been addressed within the authorship?	Most of the authors are Brazilian, including the first and last author who both have long experiences of using routine data from Brazil. QHRFF and RCOCS are PhD students. EPS, LLC, JMP and IRF are early career researchers. Almost 80% of the co-authors are women, including the first and last author.
Training	1. How has the project contributed to training of LMIC researchers?	The data produced by the Zika Platform has been used by PhD students and also early career researchers to develop their analytical skills.
Infrastructure	1. How has the project contributed to improvements in local infrastructure?	The support from this project has enabled us to hire local early career students and improve data structures that can be used by this project and others developed in the CIDACS data centre.
Governance	1. What safeguarding procedures were used to protect local study participants and researchers?	All data are currently being stored at CIDACS under strict security standards. Analyses were conducted by VPN in a trusted research environment.
